# Editorial: The broader aspects of non-alcoholic fatty liver disease in children

**DOI:** 10.3389/fped.2022.1034306

**Published:** 2022-10-06

**Authors:** Claudia Mandato, Luca Miele, Piotr Socha, Pietro Vajro

**Affiliations:** ^1^Department of Medicine, Surgery and Dentistry “Scuola Medica Salernitana”, University of Salerno, Baronissi, Italy; ^2^Department of Medical and Surgical Sciences, Policlinico Universitario A. Gemelli IRCCS Rome, Università Cattolica del S. Cuore, Rome, Italy; ^3^Department of Gastroenterology, Hepatology, Nutritional Disorders and Pediatrics, The Children's Memorial Health Institute, Warsaw, Poland

**Keywords:** non-alcoholic fatty liver disease (NAFLD), metabolic (dysfunction)-associated fatty liver disease (MAFLD), liver steatosis, liver fibrosis, cirrhosis, children

## Introduction

Non-alcoholic fatty liver disease (NAFLD), more recently proposed as metabolic (dysfunction)-associated fatty liver disease (MAFLD), is nowadays the most common chronic liver disease at all ages (1). The presence and degree of fibrosis are considered important factors in predicting the risk of developing non-alcoholic steatohepatitis (NASH) with advanced fibrosis and cirrhosis already in pediatric patients (2). Moreover, non-invasive fibrosis assessment is able to predict complications in adults (3). So far, the diagnostic gold standard remains liver biopsy, which is however expensive, invasive, and burdened by the risk of complications and sampling errors (4). To circumvent this drawback, non-invasive imaging and/or laboratory tests have become therefore the object of ever-increasing investigations to assess their role in the early detection of fibrosis. In this Research Topic, four articles examined the role of several non-invasive imaging/laboratory tests in pediatric NAFLD.

## Imaging

Yu et al. performed a systematic review and meta-analysis to evaluate the diagnostic performance of several imaging modalities for the diagnosis of fibrosis in pediatric NAFLD. Their study was based on 11 particularly robust articles (12 studies) where liver histopathology as the gold standard was compared to ultrasound (US), conventional US and US-time harmonic elastography (US-THE), Transient Elastography (TE), Shear Wave Elastography (SWE), magnetic resonance (MR), MR Elastography (MRE), and/or MR Imaging—Proton Density Fat Fraction (MRI-PDFF). MRE measured shear stiffness as a biomarker of fibrosis in pediatric non-alcoholic fatty liver disease (5).

For the diagnosis of fibrosis, US-based TE had higher sensitivity (97–100%), while MRI had the lowest sensitivity (58–63%; CI, 93–97). Regarding steatosis, the sensitivity of multi-frequency MRE-hepatic fat fraction (mMRE-HFF) was slightly better than that of MRI-PDFF and US, while the specificity of US was slightly higher than that of mMRE-HFF and MRI-PDFF. As US-based TE demonstrated the best performance in diagnosing significant fibrosis in pediatric NAFLD compared to other imaging modalities, liver stiffness measurement (LSM) with TE is expected to be a promising biological indicator for routine screening and monitoring of disease changes and prognostic evaluation.

## Serum markers of fibrosis

Three articles reported from different angles on the performance of serum markers for non-invasive assessment of pediatric liver fibrosis vs. histology or imaging.

Mosca, Volpe et al. evaluated the usefulness of four fibrosis scores [Aspartate Aminotransferase/Platelet Index (APRI), Fibrosis 4 (FIB-4), NAFLD Fibrosis Score (NFS), and Hepamet Fibrosis Score (HFS)] in predicting different degrees of fibrosis in 286 adolescents with biopsy-proven NAFLD. One hundred seventy-three patients (60.4%) had fibrosis on histological analysis. HFS (AUROC 0.778, 95% CI 0.722–0.828) and APRI (AUROC 0.619, 95% CI 0.556–0.679) performed better than NFS and FIB-4 in the distinction of subjects with fibrosis; but the respective PPV scores were not high enough to be considered diagnostic. Overall, their data indicate that these fibrosis scores cannot be used to reliably diagnose fibrosis or its course in children and cannot replace liver biopsy as the gold standard.

In another study, the same authors examined serum levels of Hyaluronic acid (HA) and N-terminal propeptide of type III procollagen (PIIINP), i.e., two known non-invasive biomarkers of increased extracellular matrix deposition (Mosca, Mantovani et al.). As the serum levels of these biomarkers may depend on renal function, possibly altered in patients with NAFLD even in childhood, the authors evaluated the association of these two biomarkers levels with estimated Glomerular Filtration Rate (eGFR; Bedside Schwartz equation) in 106 children with overweight/obesity and biopsy-proven NAFLD. They found that children with fibrosis F2 had significantly higher plasma PIIINP and HA levels than those with F0 or F1 fibrosis. Multivariate regression models showed that higher plasma PIIINP (standardized beta coefficient: −0.206, *P* = 0.011) and HA levels (standardized beta coefficient: −0.531, *P* < 0.0001) were associated with lower eGFR values, even after adjustment for age, sex, systolic blood pressure, PNPLA3 rs738409 genotype, and any stage of liver fibrosis.

Kwon et al. studied several serum biomarkers [procollagen type1 amino-terminal propeptide (P1NP), osteocalcin, interleukin-6 (IL-6), and Mac-2 binding protein glycosylated isomer (M2BPGi)] in 60 children or adolescents with advanced fibrosis and steatohepatitis as assessed by LSM and the Fibroscan-liver enzyme AST (FAST) score, respectively. IL-6 and M2BPGi levels differed between the LSM-positive and negative groups (*p*-values of 0.005 and < 0.001, respectively).

The area under receiver operating characteristic (AUROC) of IL-6 multiplied by AST values (values obtained through multivariate analysis) was 0.821 with a cut-off value of 228.15. M2BPGi showed a significant linear relationship with LSM.

The biomarker with a significant difference between the FAST-positive and negative groups was the P1NP/osteocalcin ratio (*p*-value = 0.008). The AUROC of P1NP/osteocalcin ratio multiplied by ALT values (values obtained through multivariate analysis) was 0.939 with a cut-off value of 305.38. The diagnostic capability of M2BPGi to evaluate advanced fibrosis showed an acceptable result (AUROC = 0.742; *p* = 0.022). All in all, the measurements of P1NP, IL-6, or M2BPGi along with the basic chemistry tests would help determine the stage of NAFLD at diagnosis and during follow-up.

## Discussion

Pediatric NAFLD/MAFLD has still several unresolved issues (6). Together, the articles comprising this Research Topic centered on non-invasive markers of fibrosis using serological tests and imaging techniques

- provide insights and updates into the subject of non-invasive assessment of pediatric NAFLD severity
- pinpoint the existence of several unmet needs
- help to raise a foundation for future and ongoing efforts.

Among several possible diagnostic imaging alternatives, liver stiffness measured by US-TE appears increasingly popular in children with NAFLD because it is practical, patient-friendly, and being quantitative it can also easily track disease progression.

Serum assays for products of matrix synthesis or breakdown studied over several years as surrogate markers for liver fibrosis detection in a number of hepatopathies mainly in adults (7), might now include pediatric NAFLD as well. Their performance in children, however, is still reportedly unsatisfactory because of the influence of body growth (e.g., PIIINP and procollagen IV) or bone metabolism (e.g., procollagen I). Here we have seen that HA and amino-terminal PIIINP may have a role in pediatric NAFLD, but the interference of renal function should be taken into account. Despite the statistical significance of some results, these biomarkers, unfortunately, continue hitherto to be clinically limited and therefore still of largely experimental interest (8, 9).

Fibrosis biomarkers, in adults, are allowing optimized risk stratification, thus enabling a personalized approach. The use of non-invasive biomarkers has enormous advantages compared to liver biopsy. However, they still require attention due to their prognostic significance. Data from large pediatric cohorts are indispensable to better define the role of biomarkers during the pediatric patient journey.

We thank all the Authors for their contributions dedicated to filling the existing gap with the mounting knowledge gained from adult-focused research, and eagerly look forward to the contribution also of novel strategies around the corner such as “liquid biopsy” (10).

## Author contributions

All authors listed have made a substantial, direct, and intellectual contribution to the work and approved it for publication.

## Conflict of interest

The authors declare that the research was conducted in the absence of any commercial or financial relationships that could be construed as a potential conflict of interest.

## Publisher's note

All claims expressed in this article are solely those of the authors and do not necessarily represent those of their affiliated organizations, or those of the publisher, the editors and the reviewers. Any product that may be evaluated in this article, or claim that may be made by its manufacturer, is not guaranteed or endorsed by the publisher.
